# Supplementary material for: The principles of recovery-oriented mental health services: A review of the guidelines from five different countries for developing a protocol to be implemented in Yogyakarta, Indonesia
Source: PLoS One. 2023 Mar 2;18(3):e0276802. doi: 10.1371/journal.pone.0276802 (PMC9980814; doi:10.1371/journal.pone.0276802)
Supplement: Attachment — Submitted filename: Reviewer comments.docx [file pone.0276802.s001.docx]

Dear Editor

Thanks for asking me to review this manuscript titled: “The principles of recovery-oriented mental health services: A review of the guidelines from five different countries”

The authors have written a manuscript that summarizes the theoretical background to their attempt towards developing a guideline for establishing a recovery-oriented mental health service in Indonesia. Their decision to leverage what has been implemented in other countries establishes a firm theoretical foundation for their intended program. I have no reservations in recommending that their manuscript be published as this will form a guide for other developed countries who might desire to pursue the same course.

However, I have some concerns.

Title

The title does not seem to capture the essence of this manuscript. The manuscript isn’t just about a review. It is a review of guidelines for the development of a protocol. This is also seen in the conclusion of the abstract where the authors write “We will adjust and implement the result of the review in our project focusing on developing recovery-oriented mental health service in the community health centre in Yogyakarta, Indonesia. We hope that this framework will be adopted by the central government in Indonesia and other developing countries”. I suggest that the title should be modified to reflect what this manuscript is about.

Aim

The authors stated that the aim of the review is to guide the direction of the entire project. This doesn’t suffice as a specific aim of the manuscript. If the aim is to guide the direction of the entire project, then the title should actually be about the entire project. The authors need to determine the aim of the research, and let it be in tandem with the title.

Methods

The authors stated that “We did not use formal search engines, such as Scopus, EBSCO, or ProQuest, since they mostly search for journal articles that do not have practical guidelines.” Are they sure that they would not have missed out on some studies? This might need to be stated as a limitation. Restricting the publications to those written in English should also be stated as a limitation.

A chart showing how the publications reviewed were excluded before the final selection can also be helpful.

Results

Expatiating on the results from line 182 downwards looked more like discussions which needed to be cited. There is a need to rewrite this section.

Discussion

The aim needs to be properly phrased to make it be in tandem with the discussion as it is. Otherwise, the discussion will appear to be superfluous.

Many thanks for this opportunity.
